# Editorial: Viral infections and mental health during the post-pandemic era

**DOI:** 10.3389/fpsyt.2024.1420348

**Published:** 2024-05-01

**Authors:** Jiahao Ji, Yang Zhang, Ping Wu, Jinming Han, Zhenwu Luo, Tong Zhang, Chuan Shi

**Affiliations:** ^1^ Center for Infectious Diseases, Beijing Youan Hospital, Capital Medical University, Beijing, China; ^2^ Beijing Institute of Sexually Transmitted Disease Prevention and Control, Beijing, China; ^3^ National Institute on Drug Dependence and Beijing Key Laboratory of Drug Dependence, Peking University, Beijing, China; ^4^ Department of Neurology, Xuanwu Hospital, Capital Medical University, Beijing, China; ^5^ Department of Microbiology and Immunology, Medical University of South Carolina, Charleston, SC, United States; ^6^ Clinical Research Department, Peking University Sixth Hospital, Beijing, China; ^7^ Peking University Institute of Mental Health, Sixth Hospital, Beijing, China; ^8^ National Clinical Research Center for Mental Disorders, Peking University Sixth Hospital, Beijing, China; ^9^ Key Laboratory of Mental Health, Ministry of Health, Peking University, Beijing, China

**Keywords:** mental health, viral infection, COVID-19, anxiety, depression

The outbreak of COVID-19 has prompted increased scrutiny on the correlation between viral infections and mental health (1–3). Beyond SARS-CoV-2, viral infections like HIV also have the potential to adversely affect an individual’s mental health (4). In addition to mental health issues such as anxiety and depression stemming from the physical discomfort caused by the virus, viral infections and their treatment regimens can impose psychological stressors, including worries about health status and outcomes, economic burden, and potential ostracism and discrimination (5), which in turn result in hazards to viral infection itself. As such, it is imperative to prioritize the correlation between viral infections and mental health in the post-pandemic era, and to offer holistic support and resources, encompassing both physical interventions and mental health services, to facilitate the recuperation and well-being of patients.

This Research Topic delves into the epidemiology of mental disorders in the post-pandemic era, and the relationship between mental health and viral infections, quality of life (QOL), which help us recognize the challenges that people face in terms of mental health in the post-pandemic era, and to remind us of the imperative to pay attention to mental health. Submissions included systematic reviews and meta-analysis, original research and brief research report.

## The epidemiology of depression and anxiety

The systematic review and meta-analysis by Ji et al., “*People who living with HIV/AIDS also have a high prevalence of anxiety disorders: a systematic review and meta-analysis*”, offers a thorough examination and analysis, employing stringent diagnostic criteria to determine the prevalence of anxiety disorders among people living with HIV/AIDS (PLWHA) at 15.5% and validate potential risk factors. This data is valuable for gaining insight into the mental health status of this particular population, contributing to a deeper understanding of the mechanisms involved in the onset of anxiety disorders and offering valuable insights for future research and clinical applications. The article underscores the significance of early screening and prevention of anxiety disorders in PLWHA. This serves as a call to action for clinicians and public health policymakers to prioritize the mental health of this particular demographic.

In the study entitled “*Depression and anxiety among Macau residents during the COVID-19 outbreak: A network analysis perspective*”, Sun et al. discovered a significant portion of Macau residents experienced concurrent depression and anxiety during the 6.18 COVID-19 outbreak. The network analysis conducted in the study identified central symptoms such as nervousness, uncontrollable worry, and irritability, as well as bridge symptoms like restlessness and sad mood, as potential targets for the treatment and prevention of comorbid depression and anxiety associated with this outbreak. This research offers valuable insights and recommendations for comprehending and addressing the mental health challenges of the COVID-19 pandemic through a thorough examination of comorbid symptoms, the application of an innovative network analysis approach, the identification of potential therapeutic targets, and the proposal of intervention strategies.

The original research by Leonhardt et al. entitled “*Fatal drug use in the COVID-19 pandemic response: Changing trends in drug-involved deaths before and after stay-at-home orders in Louisiana*” conducted an analysis of public health surveillance data in Louisiana before and after the implementation of a stay-at-home order during the COVID-19 pandemic. The results highlighted the need for a more strategic and coordinated response to future public health crises. This study paves the way for comprehending and addressing drug-related fatalities amidst the COVID-19 pandemic through an emphasis on public health concerns, utilization of public health surveillance data, and recognition of the increasing necessity for forthcoming interventions.

## The relationship between viral infections and mental health

In the study, “*Investigating the relationship between hepatitis B virus infection and postpartum depression in Chinese women: a retrospective cohort study*”, Huang et al. shed light on the association between maternal HBV infection and postpartum depression, prompting further investigation into the correlation between postpartum depression and HBeAg positivity, as well as previous mental health history. In conclusion, this research offers valuable insights into the relationship between HBV infection and postpartum depression, as well as the association between variables such as parity and postpartum hemorrhage. Additionally, recommendations for future investigations are provided to enhance understanding and intervention strategies for postpartum depression.

In the research entitled “*Long-term outcomes of COVID-19 intensive care unit survivors and their family members: a one year follow-up prospective study*”, Laurent et al. conducted a study examining the enduring physical, functional, and mental outcomes of COVID-19 intensive care unit (ICU) patients and their family members one year post-discharge. The research highlights the substantial global implications on the mental health of patients and their families, with a significant number reporting persistent challenges that impede their daily routines. In conclusion, this research highlights the persistent physical, functional, and psychological challenges experienced by COVID-19 patients in the intensive care unit and their families over an extended period of time. This underscores the significance of these findings for global health and underscores the necessity for enhanced support and interventions.

Collectively, these studies revealed a heightened prevalence of anxiety and depression in the post-COVID-19 pandemic era, potentially attributed to various factors. These include the post syndromes of SARS-Cov-2 infection, such as compromised respiratory function and fatigue, which are similar to symptoms of anxiety and depression. Secondly, the isolation of patients infected with SARS-Cov-2 during the outbreak may have exacerbated their depression. The interplay between physical and psychological factors may further exacerbate the incidence of anxiety and depression. Thirdly, in early stage of the epidemic, uncertainty about the future can lead to heightened anxiety. Furthermore, heightened stress levels during and after the epidemic can exacerbate feelings of anxiety and depression. Moreover, the loss of relatives during the epidemic can result in frustration and depression. Lastly, the infiltration of viral inclusions into the brain and the subsequent release of localized inflammatory factors can also contribute to the development of anxiety and depression.

## The relationship between viral infections and QOL

Peng et al. conducted a study entitled “*A network analysis of the long-term quality of life and mental distress of COVID-19 survivors 1 year after hospital discharge*”, to investigate the symptom network of mental distress and its relationship with QOL among COVID-19 survivors one year post-hospital discharge. The study identified several key symptoms that significantly contributed to mental distress and quality of life in this population. The findings suggest that early identification and targeted interventions for these symptoms could be effective in mitigating mental distress and enhancing QOL among COVID-19 survivors. This study offers valuable insights and recommendations for addressing mental health challenges among individuals who have survived COVID-19, utilizing in-depth exploration of long-term consequences, use of a network analysis approach, recommendations for early interventions, and practicality and applicability.

Wang et al.'s study, “*The relationship between social support and depression among HIV-positive men who have sex with men in China: the chain mediating role of psychological flexibility and hope*”, investigated the correlation between social support and depression among HIV-positive men who have sex with men. It was suggested that both hope and psychological flexibility act as chain mediators in this relationship, with hope also serving as a single mediator. Consequently, interventions targeting psychological flexibility and hope should be developed to improve mental health. This research offers significant contributions to the comprehension and intervention of mental health among HIV-positive homosexual individuals by conducting a thorough examination, employing diverse methodologies, uncovering insights, and discussing practical applications.

These studies offer comprehensive analyses of significant themes essential for comprehending and addressing present and future challenges in the field of viral infection and mental health. They encompass examinations of the enduring effects of the COVID-19 pandemic on individuals and families, inquiries into the mental well-being of individuals with HIV, and investigations into the correlation between social support and depression, thereby furnishing valuable perspectives and recommendations. The strength of these studies is attributed to their utilization of diverse methodologies, including network analysis, longitudinal follow-up studies, and mediation analysis, to examine their respective subjects from multiple aspects. By employing these approaches, the researchers are able to acquire a thorough comprehension of the intricate interconnections among various variables and suggest tailored interventions, thereby offering significant backing for enhancing individuals’ mental health.

Collectively, these studies are insightful in understanding the mutual influences between virus infection and mental health issues. They highlight the enduring effects and the necessity for targeted interventions to enhance support for individuals and communities. Subsequent research and practical applications should further develop upon these findings to foster enduring enhancements in mental health. Gratitude is extended to all authors who contributed to this Research Topic.

## Author contributions

JJ: Writing – original draft. YZ: Writing – original draft. PW: Writing – review & editing. JH: Writing – review & editing. ZL: Writing – review & editing. TZ: Writing – review & editing. CS: Writing – review & editing.
